# Self-Supporting Nanoporous Copper Film with High Porosity and Broadband Light Absorption for Efficient Solar Steam Generation

**DOI:** 10.1007/s40820-023-01063-z

**Published:** 2023-04-10

**Authors:** Bin Yu, Yan Wang, Ying Zhang, Zhonghua Zhang

**Affiliations:** 1 Key Laboratory for Liquid-Solid Structural Evolution and Processing of Materials (Ministry of Education), School of Materials Science and Engineering, Shandong University, Jingshi Road 17923, Jinan, 250061 People’s Republic of China; 2 School of Materials Science and Engineering, University of Jinan, West Road of Nan Xinzhuang 336, Jinan, 250022 People’s Republic of China

**Keywords:** Solar steam generation, Nanoporous copper, Broadband solar absorption, Localized surface plasmon resonance, Seawater desalination, Dealloying

## Abstract

Solar steam generation (SSG) is a potential technology for freshwater production, which is expected to address the global water shortage problem. Some noble metals with good photothermal conversion performance have received wide concerns in SSG, while high cost limits their practical applications for water purification. Herein, a self-supporting nanoporous copper (NP-Cu) film was fabricated by one-step dealloying of a specially designed Al_98_Cu_2_ precursor with a dilute solid solution structure. In-situ and ex-situ characterizations were performed to reveal the phase and microstructure evolutions during dealloying. The NP-Cu film shows a unique three-dimensional bicontinuous ligament-channel structure with high porosity (94.8%), multi scale-channels and nanoscale ligaments (24.2 ± 4.4 nm), leading to its strong broadband absorption over the 200–2500 nm wavelength More importantly, the NP-Cu film exhibits excellent SSG performance with high evaporation rate, superior efficiency and good stability. The strong desalination ability of NP-Cu also manifests its potential applications in seawater desalination. The related mechanism has been rationalized based upon the nanoporous network, localized surface plasmon resonance effect and hydrophilicity.

**Supplementary Information:**

The online version contains supplementary material available at 10.1007/s40820-023-01063-z.

## Highlights

- Self-supporting Cu film with high porosity was obtained by dealloying of Al_98_Cu_2_.
- Nanoporous Cu (NP-Cu) film shows good hydrophilicity and strong broadband light absorption.
- NP-Cu film exhibits outstanding solar steam generation and desalination performance.

**Supplementary Information:**

The online version contains supplementary material available at 10.1007/s40820-023-01063-z.

## Graphical abstract

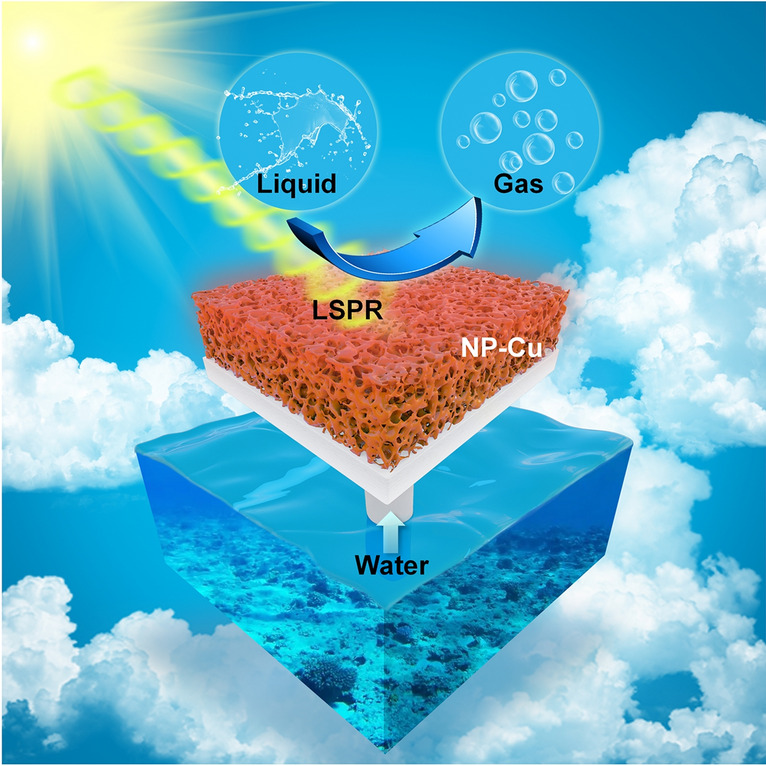

## Introduction

The shortage of freshwater resources has currently become one of the main threats to the sustainable development of human society. Unfortunately, all forms of freshwater resources are merely 2.5% of the total global water [1]. Therefore, saline water utilization is one of the important ways to solve the global freshwater shortage and has become a long-term development strategy of many countries [2]. To this end, a lot of efforts have been made to obtain clean drinking water from seawater or wastewater [3–8]. Some traditional desalination methods (such as multistage flash distillation [9] and reverse osmosis [10]) require a lot of energy [11] and pollutants generated by burning fossil fuels have a negative impact on the environment [12]. Other desalination methods such as electrodialysis and emerging capacitive deionization, which are not suitable for seawater with high salt concentrations [13, 14]. So solar steam generation (SSG) using solar energy to desalinate seawater has been considered as one of the most attractive desalination technologies [15, 16]. The SSG technology does not need any moving parts and high-pressure operation [17], which with strong expansibility is easy to be coupled with other technologies to realize multi-function such as power generation, medical sterilization and wastewater purification [18–21].

Efficient SSG systems need to meet the following characteristics: excellent photothermal conversion capability, reasonable heat management, and efficient water transportation and evaporation. Photothermal materials with excellent solar absorption and photothermal conversion capacities are the basis to ensure efficient SSG [22]. Over the past decade, many SSG systems have been developed based on different types of photothermal materials, such as metals [23–25], semiconductors [26–28], polymers [29–31], biomass carbon materials [32–34], MXenes [35], and graphenes [36–38]. Among them, plasmonic metal nanomaterials (Au [39–41], Ag [42–44], Pd [45, 46], Pt [47, 48], etc.) have aroused extensive attention due to their localized surface plasmon resonance (LSPR) property. Compared with other photothermal materials, plasmonic metal nanomaterials have the advantages of easy control of optical properties and structure, adjustable heat radiation loss, abundant optional types and good mechanical stability [49, 50]. However, the cost and practicability of noble metals hinder their further applications in SSG. Hence, developing non-noble metal-based photothermal materials is of central importance to achieve efficient and durable SSG [51].

Photothermal materials have good hydrophilicity and sufficient porous structure by constructing hierarchical nano/microstructures, which is an important guarantee for high-efficiency sunlight absorption, water transfer and steam escape [52]. Traditional metal-based photothermal films are obtained by depositing metal nanoparticles onto porous substrates (such as airlaid paper, wood, filter paper and carbon cloth) [42]. It would be better to construct metal-based photothermal films with a porous structure and self-supporting morphology. Dealloying during which the more noble element diffuses and reorganizes into a three-dimensional (3D) bicontinuous ligament/channel structure with the selective removal of the less noble element, has been widely used to prepare nanoporous metals [53, 54]. This provides an idea for preparing self-supporting porous metal-based photothermal films. For example, Zhang et al. [39] reported a dealloying-driven black gold film with a hierarchically porous structure and good SSG performance. Cu is a potential candidate for photothermal materials because of its good plasmonic properties and low cost [55]. Nanoscale Cu is a typical plasmonic metal and the potential application of Cu in SSG has been explored [56–59]. However, nanoporous Cu (NP-Cu) films fabricated by dealloying have received less attention in SSG.

Herein, we demonstrated a one-step dealloying strategy to fabricate a self-supporting NP-Cu film with high porosity and light weight. A dilute solid solution alloy (Al_98_Cu_2_, at%) was elaborately designed for dealloying, and the involved microstructural evolutions were probed by in-situ and ex-situ characterization methods. The obtained NP-Cu film with the porosity of 94.8% and density of 0.4679 g cm^–3^ shows excellent broadband light absorption of 200–2500 nm in wavelength and outstanding SSG performance.

## Experimental

### Materials Preparation

The Al_98_Cu_2_ ingot was prepared by co-melting Al and Cu (99.99 wt% purity) in a sealed quartz tube filled with argon using high-frequency induction heating. Then the ingot was cold-rolled to a sheet with a thickness of around 300 µm. Afterwards, the sheet was annealed at 550 °C for 300 min in vacuum and then immediately quenched in water. Eventually, the as-quenched Al_98_Cu_2_ sheet was dealloyed in a 0.5 M NaOH aqueous solution until no gas bubbles evolved at room temperature, and the self-supporting NP-Cu film was obtained. The dealloying process usually lasted for about 11 h. Additionally, the NP-Cu film was annealed in an argon-hydrogen atmosphere at 500 °C for 120 min to prepare the coarsened sample (named as NP-Cu-500).

### Materials Characterization

The phase compositions of all samples were probed by X-ray diffraction (XRD, XD-3) with Cu Kα radiation. The microstructures and chemical compositions of the as-dealloyed samples were characterized by transmission electron microscopy (TEM, FEI Titan 80–300) and scanning electron microscopy (SEM, JSM-7800F) equipped with an energy dispersive X-ray (EDX) analyzer. Electron backscattering diffraction (EBSD) analysis of the Al_98_Cu_2_ precursor was also performed using SEM. X-ray photoelectron spectroscopy (XPS) was used to characterize the chemical states of elements in the dealloyed samples using an AXIS Supra spectrometer with Al Kα exciting source. All XPS spectra were calibrated by C 1*s* with the binding energy at 284.6 eV. Absorption spectra of the NP-Cu/NP-Cu-500 films were recorded by employing an ultraviolet-visible-near-infrared (UV-vis-NIR) spectrophotometer (UV-3600, Shimadzu) equipped with an integrating sphere. The infrared reflection spectrum of the NP-Cu film was measured by a Fourier transform infrared (FTIR) spectrometer (Nicolet iS50). The thermal conductivities were measured by a hot disk method (Hot Disk TPS 2500S). The DSA100S goniometer was used to measure the contact angle. In addition, in-situ XRD and ex-situ SEM were conducted to explore the phase and microstructure evolutions of the Al_98_Cu_2_ precursor during dealloying.

### SSG and Desalination Experiments

Figure S1 shows the SSG setup. A solar simulator (PLS-SXE300/300UV) with an AM 1.5G filter was used as the light source. An optical power meter (PL-MW2000) was used to detect the light intensity. Infrared images and the corresponding temperatures were recorded by an IR camera (FLIR E8xt). The real-time mass change of water was recorded by an electronic balance (BSA124S-CW, Sartorius). A SSG system with a wick structure was used to test the water evaporation capacity of the NP-Cu samples. The samples were placed on a polystyrene (PS) foam, and a cotton pillar was used as a channel to supply water. The SSG tests were performed at 28 °C and relative humidity of about 40%. Additionally, the desalination ability of NP-Cu was tested using natural seawater from Bohai Sea, South China Sea and Yellow Sea. The ion concentrations in the seawater and the collected clean water were determined by inductively coupled plasma mass spectrometer (ICP-MS, Agilent 7700).

## Results and Discussion

### Fabrication and Structural Characterization of NP-Cu Films

**Fig. 1:**
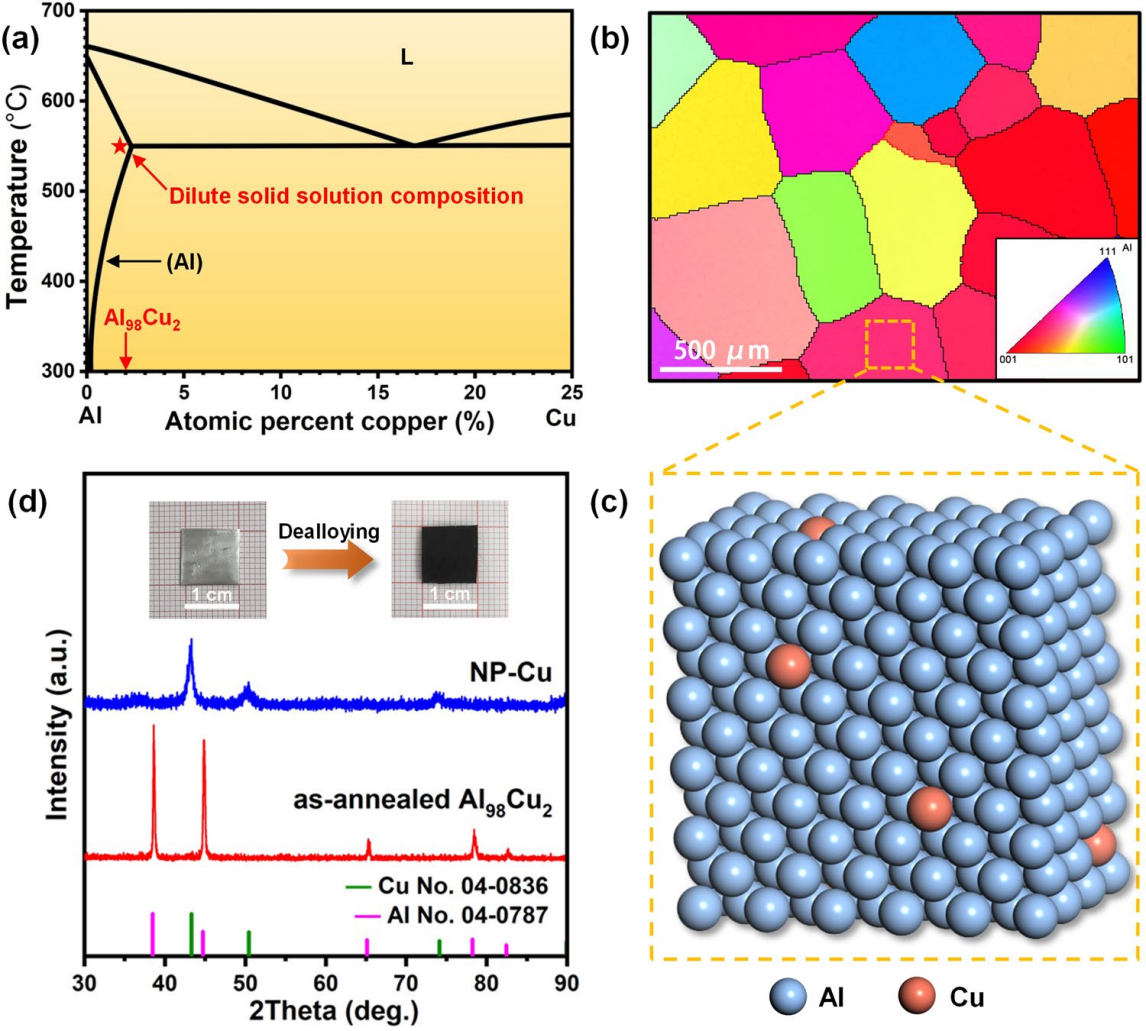
**a** Phase diagram of Al-Cu indicating the design of dilute solid solution alloy. **b** EBSD image (Inset: corresponding crystallographic orientation) and **c** schematic diagram of the crystal structure of the Al_98_Cu_2_ precursor. **d** XRD patterns (Inset: photographs of the samples before and after dealloying) of the as-annealed Al_98_Cu_2_ precursor and the NP-Cu film

According to the phase diagram of Al-Cu (Fig. 1a) [60, 61], the composition point of the precursor was set as 2 at%. The annealing and quenching treatments could ensure the formation of solid solution in the Al_98_Cu_2_ precursor. The EBSD image (Fig. 1b and inset) clearly reveals the size (several hundred microns), shape and crystallographic orientations of equiaxed grains in the Al_98_Cu_2_ precursor. Due to the minor content of Cu in Al_98_Cu_2_, Cu atoms can occupy the lattice sites of Al to form the dilute Al(Cu) solid solution (Fig. 1c). As shown in Fig. S2, the as-rolled Al_98_Cu_2_ precursor is composed of Al phase (PDF# 04-0787) and minor Al_2_Cu phase (PDF# 02-1309). In comparison, the as-annealed sample only consists of a single Al phase (Fig. 1d), indicating the formation of Al(Cu) solid solution. After dealloying, the XRD pattern of the NP-Cu film (Fig. 1d) only shows three broad peaks (at 2θ = 43.3°, 50.4° and 74.1°) of the Cu phase (PDF# 04-0836), indicating the thorough dealloying of Al_98_Cu_2_ in the NaOH solution. The EDX results (Fig. S3) further confirm that most of Al was selectively etched away during dealloying and the residual Al amount is only 1.3 at%. Moreover, the color of the sample changed from silvery white (Al_98_Cu_2_) to black (NP-Cu) after dealloying, but its self-supporting characteristic is well retained (inset of Fig. 4d).

**Fig. 2:**
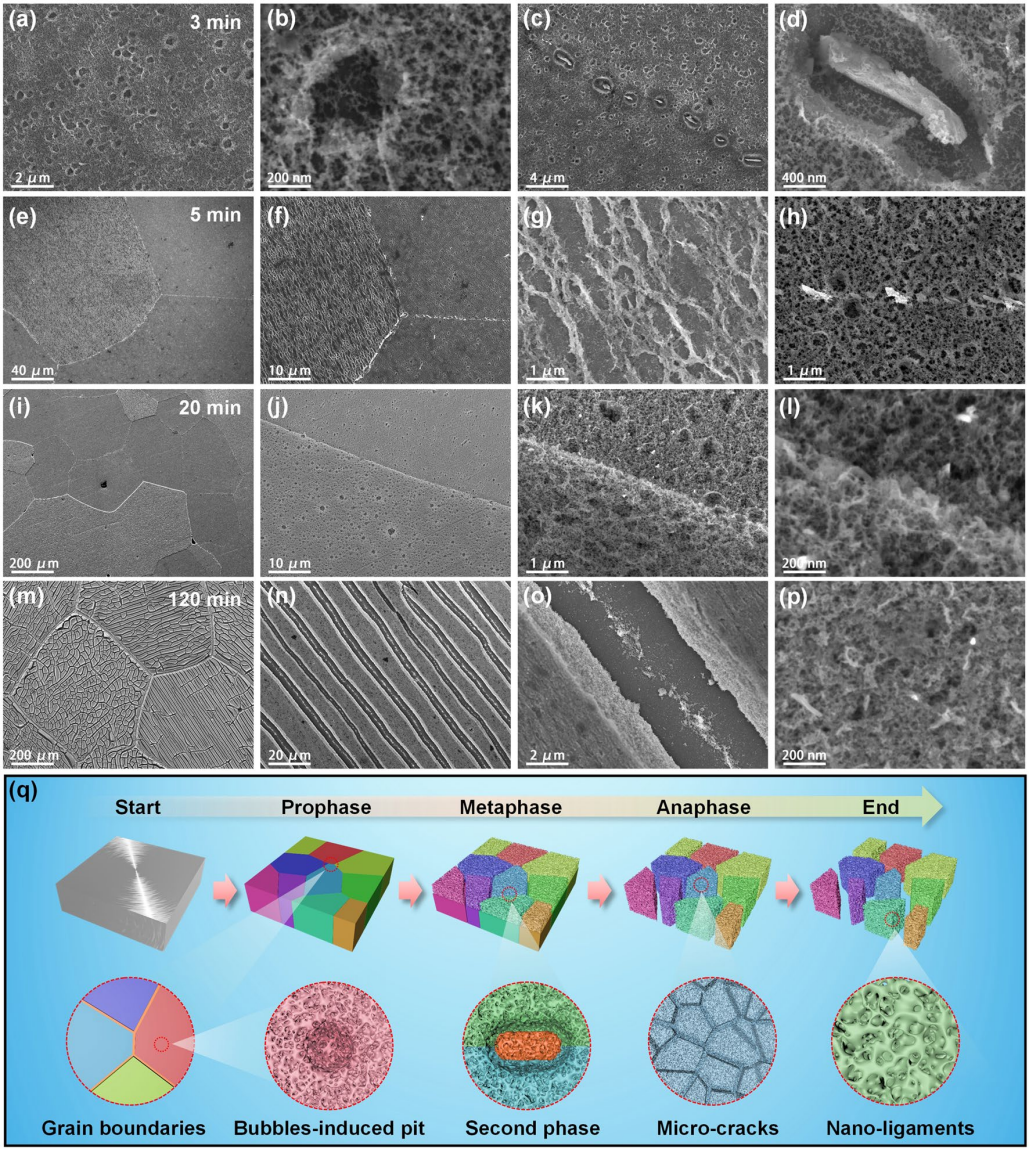
Plan-view SEM images of Al_98_Cu_2_ dealloyed for (**a–d**) 3, (**e–h**) 5, (**i–l**) 20, and (**m–p**) 120 min in the 0.5 M NaOH solution. **q** Schematic illustrations showing the microstructure evolution of Al_98_Cu_2_ during dealloying

The microstructural evolution of the Al_98_Cu_2_ precursor during dealloying was further explored by ex-situ SEM (Figs. 2 and S4). After 1 min of dealloying, the surface of Al_98_Cu_2_ was slightly corroded (Fig. S4a) and island-like humps formed (Fig. S4b). Notably, some irregular second-phase (Al_2_Cu) particles appeared inside corrosion pits along the grain boundaries (Fig. S4c, d), but could not be detected by XRD (Fig. 1d). After 3 min of dealloying, bubbles-induced pits emerged on the sample surface and the bicontinuous ligament-channel structure could be clearly observed (Fig. 2a, b). Moreover, the corrosion degree of the grain boundaries deepened (Fig. 2c, d). The grains and grain boundaries became more obvious with increasing dealloying time to 5 min (Fig. 2e, f). Furthermore, different grains showed distinct corrosion characteristics (Fig. 2g, h). Notably, some grain surfaces appear fibrous structures after 5 min of dealloying (Fig. 2e–g), probably due to different orientations of the grains (Fig. 1b). With the extension of dealloying time, the surface corrosion further deepened, and the nanoporous structure became more obvious (Fig. S4e–h). After 20 min of dealloying (Fig. 2i), the grain shape and size of the dealloyed surface are similar to those in the precursor (Fig. 1b), and the second phase at the grain boundaries disappeared owing to the dealloying of Al_2_Cu (Fig. 2j–l). When dealloying for 60 min, farmland-like cracks appeared inside the grains (Fig. S4i–l). After 120 min of dealloying, both interlaced and parallel cracks appeared in different grains, and the ligament-channel structure could be observed in the porous layer (Fig. 2m–p). The photographs in Fig. S5 show the gradual blackening of the surface of the dealloyed samples. Figure 2q vividly demonstrates the structural characteristics and evolution of different dealloying stages.

**Fig. 3:**
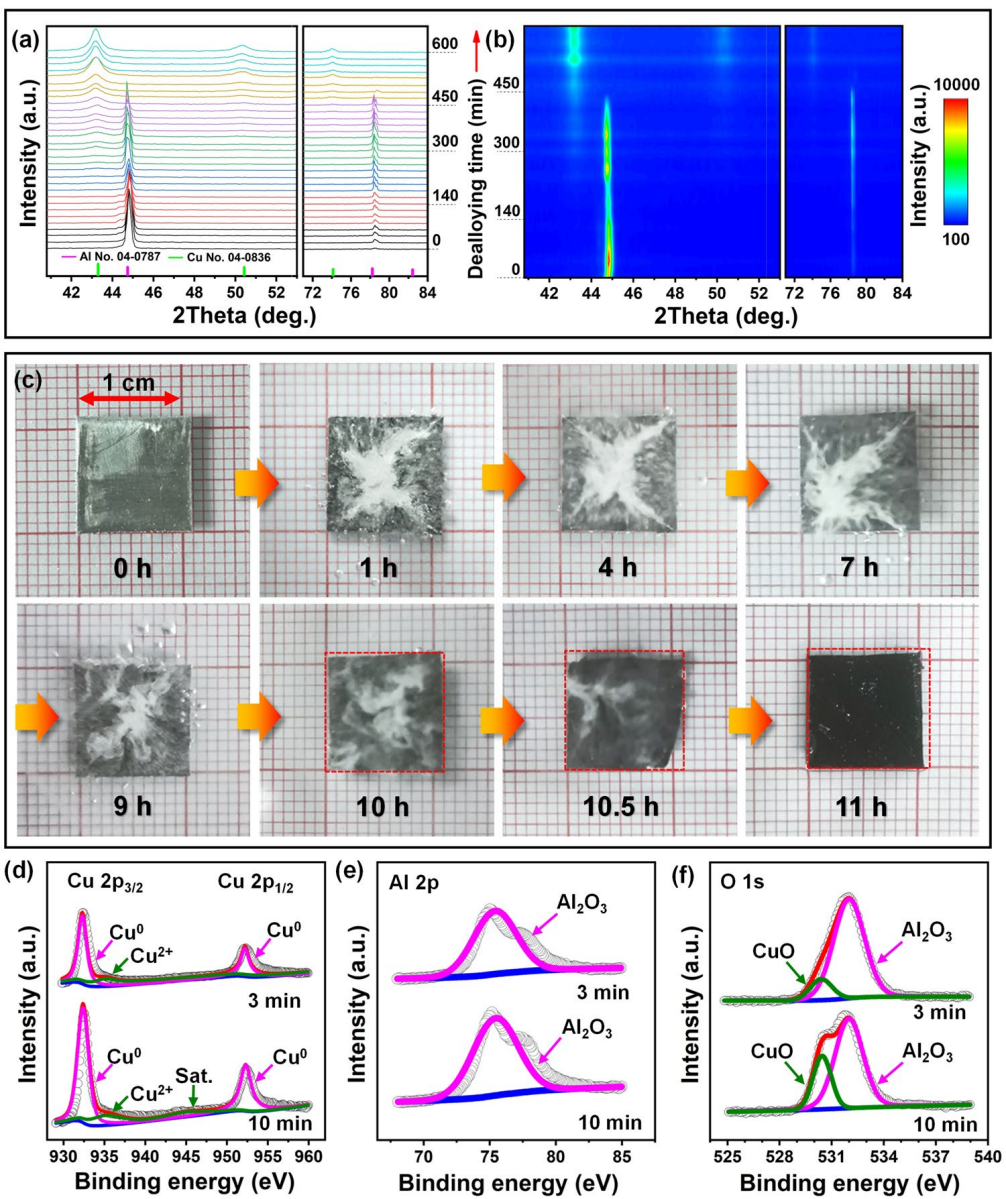
**a** In-situ XRD patterns and **b** corresponding contour plot showing the phase evolution of the Al_98_Cu_2_ precursor during dealloying in the 0.5 M NaOH solution. **c** Macrophotographs of the Al_98_Cu_2_ alloy foil dealloyed for different durations. The red dashed boxes represent the size of the pristine precursor. XPS spectra of **d** Cu 2*p*, **e** Al 2*p* and **f** O 1*s* of the Al_98_Cu_2_ foils dealloyed for 3 and 10 min

In-situ XRD was further performed to explore the phase evolution during dealloying of Al_98_Cu_2_ (Fig. 3a, b). Figure 3a shows that the peak intensity of Al(Cu) gradually decreases with the prolongation of the dealloying time. The broad diffraction peak of Cu (111) begins to appear after 300 min of dealloying and gradually becomes stronger. Thereafter, the other two diffraction peaks of Cu (200) and (220) can be observed, whose intensities further increase with dealloying time. But their peak positions do not change with time, and are consistent with standard values of *f.c.c.* Cu. And no intermediate phase emerges during the whole dealloying process. Finally, only Cu peaks remain in the XRD pattern. The corresponding contour plot in Fig. 3b visually shows the phase evolution and involved strength/position changes of diffraction peaks with the dealloying time. Figure 3c shows the macroscopic morphology/color change of Al_98_Cu_2_ during dealloying. Violent H_2_ bubbles formed due to the reaction of Al with NaOH before 9 h of dealloying. Subsequently, the bubbles obviously decreased and finally disappeared at the dealloying of 11 h. And the color of the sample became dark and black. In addition, after 10.5 h of dealloying, an evident area shrinkage (ΔS/S_0_) could be observed, and the final area shrinkage is around 14%.

The composition and valence state of the Al_98_Cu_2_ samples dealloyed for 3 and 10 min were further determined by XPS. Two peaks located at 932.4 and 952.3 eV of Cu 2*p* spectra (Fig. 3d) can be assigned to the Cu 2*p*_3/2_ and Cu 2*p*_1/2_ signals, respectively, illustrating the existence of the metallic state (Cu^0^) [62, 63]. Meanwhile, the peak of Cu^2+^ in CuO and its shake-up satellite peak can be observed at 935.4 and 945.0 eV, respectively, indicating the slight surface oxidation. When the dealloying time was extended from 3 to 10 min, the peak intensity of Cu^0^ increases obviously. This is because Al atoms on the surface of Al_98_Cu_2_ were constantly corroded away and the nanoporous structure composed of Cu formed. The Al 2*p* spectra (Fig. 3e) show a characteristic peak at 75.4 eV, indicating the presence of Al^3+^ (Al_2_O_3_) [64]. The peak intensity of Al 2*p* has no obvious change for these two samples. Additionally, the O 1*s* peaks are composed of two components (Fig. 3f). The two peaks at 530.4 [63] and 532.0 [65, 66] eV correspond to CuO and Al_2_O_3_, respectively. Compared with the scenario of 3 min of dealloying, the peak intensity associated with CuO is significantly enhanced at dealloying for 10 min, which is related to the increase of Cu content on the sample surface.

**Fig. 4:**
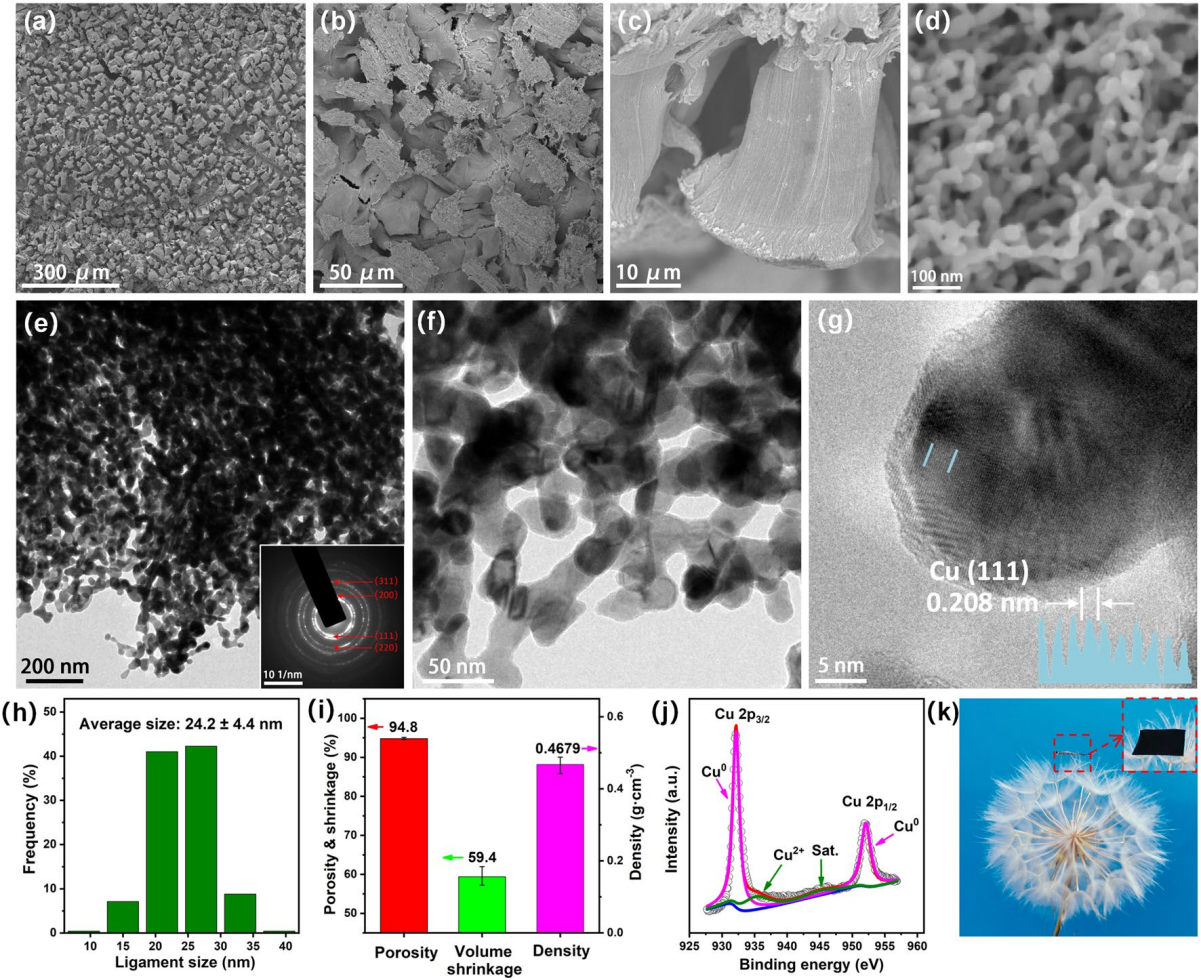
**a–d** SEM images, **e**, **f** TEM images (Inset: SAED pattern), **g** HRTEM image, and **h** ligament size distribution of the NP-Cu film. **i** Summary of porosity, volume shrinkage and density, **j** XPS spectrum of Cu 2*p*, and **k** photograph of the NP-Cu film

The obtained NP-Cu film was further characterized by SEM and TEM (Figs. 4a–g and S6). The SEM images in Figs. 4a, b and S6a show the river bed-like morphology of the NP-Cu film surface, which displays numerous channels with tens of microns in width. The cross-sectional SEM images in Figs. 4c and S6d–f show that the channels run through the whole section of NP-Cu and can serve as effective paths for water transport. Figure 4d displays a typical 3D bicontinuous ligament-channel structure of the NP-Cu film with the average ligament size of 21.9 ± 3.6 nm (Fig. S7a). The representative TEM images (Fig. 4e, f) reveal the typical nanoporous structure of NP-Cu, and nanoscale pores/ligaments can be visualized. The selected-area electron diffraction (SAED) pattern of NP-Cu (inset of Fig. 4e) reveals polycrystalline rings which can be indexed as (111), (200), (220) and (311) planes of the *f.c.c.* Cu (in agreement with the XRD result in Fig. 1d). Figure 4g illustrates the high-resolution TEM (HRTEM) image with lattice fringes of Cu (111). Based on the TEM results, the average ligament size of NP-Cu was further determined to be 24.2 ± 4.4 nm (Fig. 4h). Noticeably, the NP-Cu film possesses an ultrahigh porosity of 94.8% (Section S2), which is caused by a large number of microscale channels and nanoscale pores produced by the dealloying of the dilute solid solution Al_98_Cu_2_ (Fig. 4i). The volume shrinkage is about 59.4%, which is compatible with the obvious area shrinkage and huge thickness shrinkage (Figs. 3c and S7b). In addition, the density of the NP-Cu film is only 0.4679 g cm^–3^, much lower than that of bulk Cu (8.960 g cm^–3^).

XPS was applied to further analyze the chemical valences of the NP-Cu film (Figs. 4j and S8) and the Al_98_Cu_2_ precursor (Fig. S9). In the NP-Cu film, the binding energies at 932.2 and 952.1 eV are assigned to Cu^0^, while the peaks located at 935.2 and 945.0 eV are attributed to the Cu^2+^ and corresponding satellite peak, respectively (Fig. 4j) [62, 63]. The weak peaks of Cu^2+^ indicate that the NP-Cu film was slightly oxidized. However, the signal of Cu 2*p* in the Al_98_Cu_2_ precursor is weak, and only weak peaks of Cu^0^ at 932.6 and 952.5 eV can be observed (Fig. S9a). This is due to the extremely low Cu content in the dilute solid solution alloy. The XPS spectrum of Al 2*p* in Fig. S8b also confirms the minor residual of Al in the NP-Cu film in the state of Al^3+^ (Al_2_O_3_) [64]. In the Al_98_Cu_2_ precursor, the peaks of metallic state (Al^0^) and oxidation state (Al^3+^) of Al are located at 73.1 and 74.7 eV, respectively (Fig. S9b) [64]. In contrast, the peak intensity of Al^3+^ is higher, indicating that Al on the surface of the Al_98_Cu_2_ precursor is easy to be oxidized in air. The XPS spectrum of O 1*s* in Figure S8c shows two peaks at 530.2 and 531.9 eV in the NP-Cu film, corresponding to CuO [63] and Al_2_O_3_ [65, 66], respectively. Compared with the scenario of 3 and 10 min of dealloying, the relative peak intensity of CuO is further increased. This is the result of the thorough dealloying, which leads to a great decrease in Al content and a significant increase in Cu content. While in the Al_98_Cu_2_ precursor, only the peak associated with Al_2_O_3_ at 531.8 eV can be observed (Fig. S9c). Notably, dandelion fluff can support the NP-Cu film (Fig. 4k), which intuitively shows the advantages of light weight. Additionally, the NP-Cu-500 film is still composed of *f.c.c.* Cu, and the color changed from black to dark red and the size obviously shrank after annealing (Fig. S10a). The nanoporous structure coarsened to 47 ± 11 nm (Fig. S10b, c).

### SSG Performance of NP-Cu Films

**Fig. 5:**
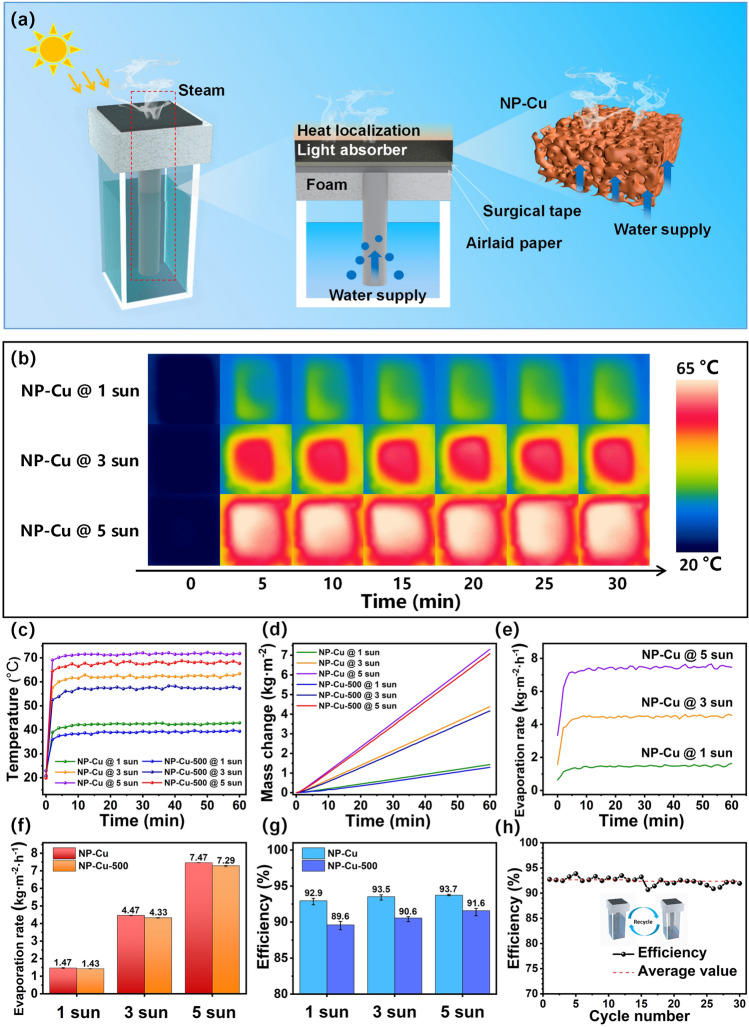
**a** Schematic illustration of water evaporation process and mechanism. **b** Infrared images, **c** surface temperature changes, **d** mass changes, **e** evaporation rate with time, **f** evaporation rate and **g** evaporation efficiency of the NP-Cu and NP-Cu-500 films under different illuminations. **h** Cycling test of the NP-Cu film under 1 sun illumination

The SSG performance of the NP-Cu and NP-Cu-500 films was thus evaluated and the evaporator schematic is shown in Fig. 5a. The NP-Cu film can not only absorb sunlight and release heat through the LSPR effect, but also transport water through internal multi-scale channels. The PS foam with low thermal conductivity (0.04 W m^–1^ K^–1^) can isolate the unnecessary heat exchange between the SSG system and the surrounding environment. Figure 5b–h shows the water evaporation performance of the NP-Cu film, with the NP-Cu-500 film as the benchmark. Infrared images (Fig. 5b) show the surface temperature distribution of the NP-Cu film under various illuminations and times. Regardless of the light intensity, the surface temperature of NP-Cu rapidly rises and remains stable thereafter. Figure 5c illustrates the time-dependent temperature changes. The surface temperature of NP-Cu increases sharply within 5 min and then reaches the plateau with slight temperature fluctuation under various illuminations. This phenomenon suggests that the NP-Cu film possesses good photothermal conversion capability. Specifically, the maximum surface temperatures of NP-Cu can reach up to 42.4, 62.3 and 71.6 °C under 1, 3 and 5 sun illumination, respectively. In comparison, the surface temperature of the NP-Cu-500 film can also increase rapidly and then keep a stable plateau of 39.1, 57.5 and 68.1 °C under 1, 3 and 5 sun illumination, respectively, lower than that of NP-Cu at the same illumination. This difference can be attributed to the fact that the smaller ligaments of NP-Cu can enrich more free electrons on the surface, thus enhancing the LSPR effect [67]. Figure 5d illustrates the mass change curves of the two films. The final mass changes of NP-Cu are 1.41, 4.31 and 7.18 kg m^–2^ under 1, 3 and 5 sun illumination respectively. Meanwhile, the mass changes of NP-Cu-500 are slightly smaller than those of NP-Cu under different illuminations. Figure 5e displays the curves of evaporation rate with time of NP-Cu. Apparently, the evaporation rate rises rapidly within 5 min and then remains stable. Figure 5f compares the evaporation rates of the NP-Cu and NP-Cu-500 films. The evaporation rate of NP-Cu is 1.47 kg m^–2^ h^–1^ under 1 sun illumination, slightly greater than that of NP-Cu-500 (1.43 kg m^–2^ h^–1^). Under the light intensity (5 sun), the NP-Cu film shows the highest evaporation rate (7.47 kg m^–2^ h^–1^), still higher than that of NP-Cu-500 (7.29 kg m^–2^ h^–1^). The evaporation efficiency (η) was calculated by the following equation [68]:

1 $$\eta = \frac{{\dot{m}h_{LV} }}{I}$$

where $\dot{m}$ represents the evaporation rate in equilibrium, $h_{LV}$ is the total enthalpy of liquid-vapor phase change (2260 kJ kg^–1^) [69], $I$ represents the power density of incident light. Figure 5g shows the evaporation efficiencies of the NP-Cu and NP-Cu-500 films. The evaporation efficiencies of the two films slightly fluctuate under different light intensities. The evaporation efficiencies of NP-Cu are 92.9%, 93.5% and 93.7% under 1, 3 and 5 sun illumination respectively, higher than those (89.6%, 90.6% and 91.6%) of NP-Cu-500. The evaporation efficiencies of NP-Cu and NP-Cu-500 films fluctuate little in 30 cycles (Figs. 5h and S11), indicating that the SSG system has good stability and durability. Moreover, there is no obvious change in the ligament size of NP-Cu after the cycling test (Fig. S12), suggesting the good structural stability of NP-Cu even under the irradiation of sunlight.

### Seawater Desalination Property

**Fig. 6:**
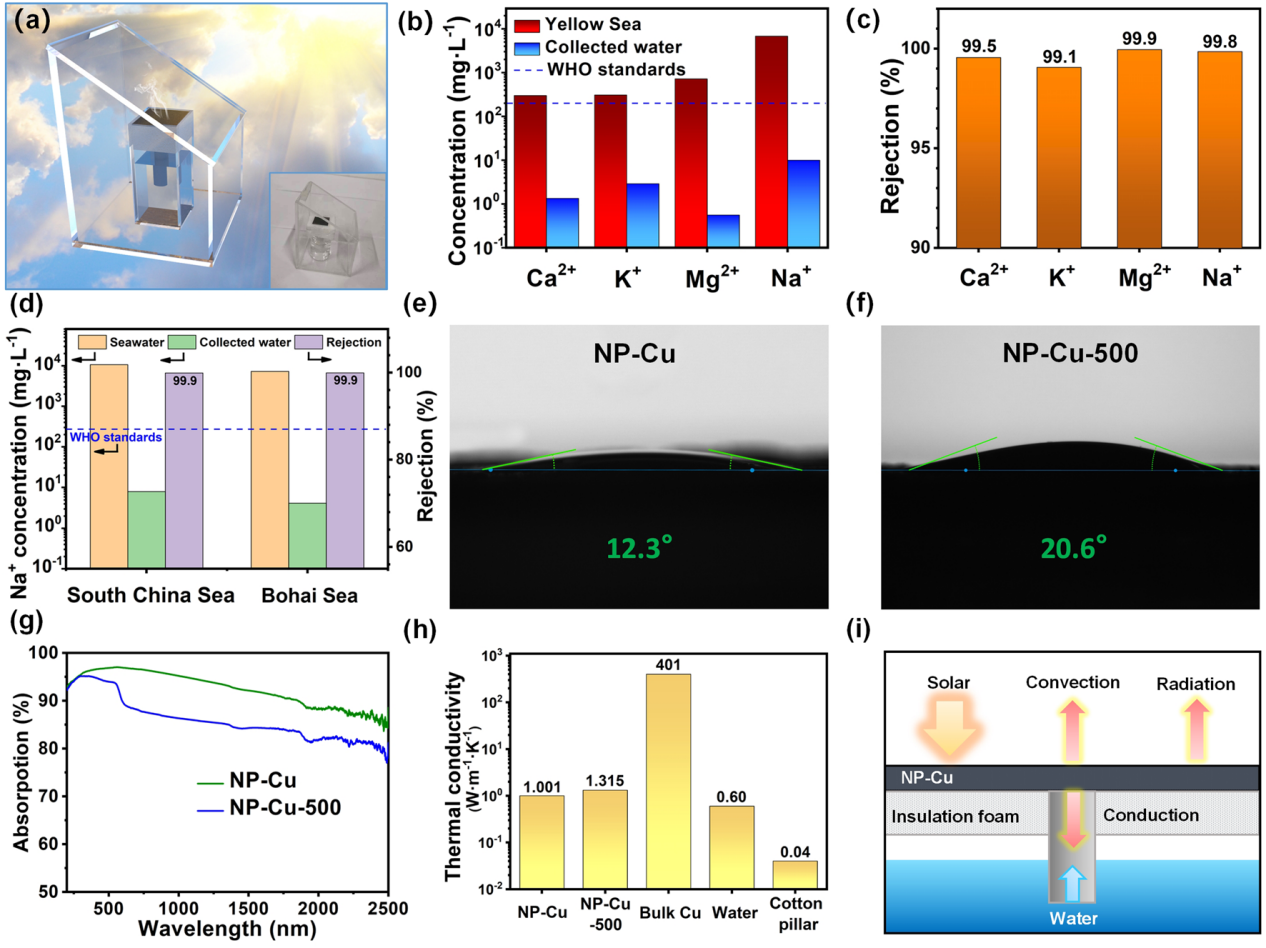
**a** Schematic illustration of steam condensation recovery device. Inset: photograph of the device. **b** Concentrations of four metal ions in Yellow Sea and the collected clean water after desalination by the NP-Cu film. **c** Ion rejection of real seawater sample after desalination. **d** Na^+^ concentrations in seawater (South China Sea and Bohai Sea) and the collected clean water after desalination by the NP-Cu film and corresponding rejection. **e**, **f** Contact angles of the **e** NP-Cu and **f** NP-Cu-500 films. **g** UV-vis-NIR absorption spectra of the NP-Cu and NP-Cu-500 films. **h** Summary of thermal conductivities for some related materials. **i** Schematic diagram of heat losses

In order to verify the seawater desalination performance of NP-Cu, a simple condensation recovery device was made for water purification (Fig. 6a). Water vapor escaping from the surface of the SSG system can condense on the inclined plane and then be collected. As observed in Fig. 6b, the ion concentration of Ca^2+^, K^+^, Mg^2+^ and Na^+^ in the seawater (Yellow Sea) decreases from 298.7, 307, 716, 6765 to 1.3, 2.9, 0.6, 9.9 mg L^–1^ respectively, which is noticeably reduced after desalination and completely satisfies drinking water standards of the World Health Organization (WHO) [70]. Meanwhile, the NP-Cu film exhibits high ion rejections of more than 99.1% (Fig. 6c). Moreover, desalination experiments of different seawater (Bohai Sea and South China Sea) further prove the compatibility and adaptability of NP-Cu (Fig. 6d). These results jointly indicate the potential of the present NP-Cu film for seawater desalination applications. Similarly, the NP-Cu-500 film also shows good seawater desalination ability (Fig. S13). In order to study the influence of salt accumulation on the NP-Cu film, the seawater was used for solar evaporation cycle tests under 1 sun illumination. As shown in Fig. S14a, the evaporation efficiency of the NP-Cu film decreases slightly from 92.1% after 1 h of illumination to 87.9% after 7 h of illumination. The evaporation rate of the NP-Cu film has a similar downward trend. Obviously, compared with the initial state (Fig. S14b), salt accumulation appeared on the surface of the NP-Cu film after 7 h of illumination (Fig. S14c). The salt accumulation can block the porous structure of the surface, thus hinder water transfer and reduce light area, resulting in the decrease of the SSG performance of the NP-Cu film [71]. However, some salt crystals re-dissolved without illumination for 1 h (Fig. S14d), indicating the NP-Cu film has a certain anti-salt fouling ability.

### Mechanism Analysis

The intrinsic mechanism of good SSG performance of NP-Cu was further explored. Excellent hydrophilicity is an important condition for efficient evaporation of photothermal materials [72, 73]. The contact angle will be affected by the surface pore structure and its distribution [74]. The contact angles of NP-Cu and NP-Cu-500 are 12.3° and 20.6°, respectively (Fig. 6e, f), indicative of their excellent wettability and good water storage capacity. This means that water supply is not the key factor causing the difference of their SSG performance. To evaluate the light absorption capacity of the NP-Cu film and the NP-Cu-500 film, Fig. 6g shows the UV-vis-NIR spectra of NP-Cu and NP-Cu-500 in the wavelength range of 200–2500 nm. As seen in the whole spectra, the light absorption of NP-Cu is higher than that of NP-Cu-500. The high light absorption of NP-Cu-500 is mainly concentrated in the visible light region. In comparison, the NP-Cu film exhibits the good broadband absorption across the whole spectrum range. Especially in the wavelength range of 276–1039 nm, its absorption is more than 95%. The 3D bicontinuous ligament-channel structure as well as the fine ligaments (24.2 ± 4.4 nm) of NP-Cu is beneficial to increase the scattering path, thus enhancing light absorption [75]. Low thermal conductivity is a necessary condition for an ideal SSG system [26, 76], which can effectively reduce heat loss. The thermal conductivity of the NP-Cu film (1.001 W m^–1^ K^–1^) or the NP-Cu-500 film (1.315 W m^–1^ K^–1^) is much smaller than that of bulk Cu (401 W m^–1^ K^–1^) and slightly larger than that of water (0.60 W m^–1^ K^–1^) (Fig. 6h). The low thermal conductivity can efficiently localize the generated heat at the evaporation surface of the SSG system and avoid the rapid heat loss to the environment [77]. And the PS foam and cotton pillar with extremely low thermal conductivity (0.04 W m^–1^ K^–1^) are beneficial to reduce the downward heat loss. Figure 6i reveals three main ways of heat loss, including conduction, convection and radiation [17, 78]. The heat conduction loss only accounts for 0.13% (Section S3), which further illustrates that the SSG system owns good thermal management and can make full use of heat to improve the evaporation efficiency.

Thus the excellent SSG performance of NP-Cu can be rationalized as follows. The unique 3D bicontinuous network structure of the NP-Cu film, coupled with multi-scale local structures (such as nano-ligaments and micro-channels), can effectively achieve broadband absorption of light [79–83]. The rough surface and the porous structure can enable multiple reflections, so as to improve light absorption. The generated steam can quickly escape from the porous structure of the NP-Cu film. Besides, the fine nano-ligament structure is beneficial to enhance the LSPR effect, thus achieving favorable photothermal conversion ability. The low thermal conductivity of each part of the SSG system contributes to realizing a stable heat concentration, limiting the converted heat to the photothermal layer and reducing heat loss. The good hydrophilicity makes the NP-Cu film have excellent water storage and delivery capability. Due to these good properties, the NP-Cu film possesses excellent SSG performance, coupled with low metal cost (Table S1), which has great development potentials as benchmarked with noble metals-based photothermal materials.

## Conclusions

In summary, the self-supporting NP-Cu film with high porosity (94.8%) can be fabricated by one-step dealloying of the dilute solid Al_98_Cu_2_ precursor in the alkaline solution. The in-situ XRD and ex-situ SEM/XPS results well reveal the phase/microstructure/composition evolutions during the dealloying of Al_98_Cu_2_. The unique 3D network structure and multi-scale channels endow the NP-Cu film with good broadband absorption capability. The NP-Cu film exhibits excellent SSG performance (evaporation rate, efficiency and stability) and seawater desalination capability, which is associated with its broadband light absorption, enhanced LSPR effect by the nanoscale ligaments and good hydrophilicity. Due to the low price of Cu compared with precious metals like Au and Ag, this work provides a new approach to the design and fabrication of low-cost metal-based photothermal conversion materials for SSG systems.

### Supplementary Information

Below is the link to the electronic supplementary material.

Supplementary file1 (PDF 1147 KB)
