# Supplementary material for: Self-Supporting Nanoporous Copper Film with High Porosity and Broadband Light Absorption for Efficient Solar Steam Generation
Source: Nanomicro Lett. 2023 Apr 10;15:94. doi: 10.1007/s40820-023-01063-z (PMC10086088; doi:10.1007/s40820-023-01063-z)
Supplement: Supplementary file 1 — Supplementary file1 (PDF 1147 KB) [file 40820_2023_1063_MOESM1_ESM.pdf]

Supporting Information for

Self-Supporting Nanoporous Copper Film with High Porosity and Broadband Light Absorption for Efficient Solar Steam Generation

Bin Yu[1], Yan Wang[2], Ying Zhang[1], Zhonghua Zhang[1, *]

[1] Key Laboratory for Liquid-Solid Structural Evolution and Processing of Materials (Ministry of Education), School of Materials Science and Engineering, Shandong University, Jingshi Road 17923, Jinan 250061, P. R. China

[2] School of Materials Science and Engineering, University of Jinan, West Road of Nan Xinzhuang 336, Jinan 250022, P. R. China

*Corresponding author. E-mail: zh_zhang@sdu.edu.cn (Z. Zhang)

S1 Supplementary Figures and Table

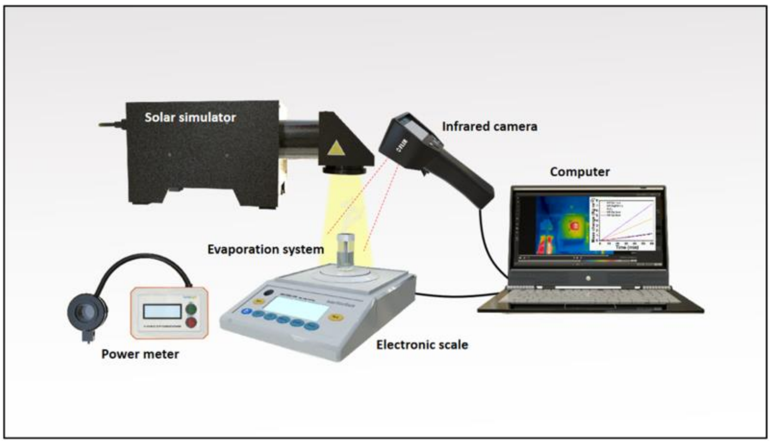

Fig. S1 Schematic of the SSG setup

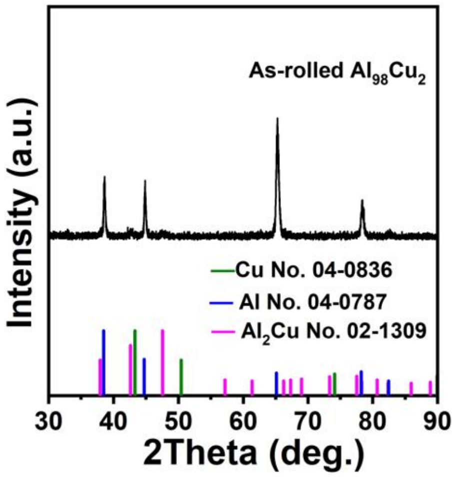

Fig. S2 XRD pattern of the as-rolled $Al_{98}Cu_2$ precursor

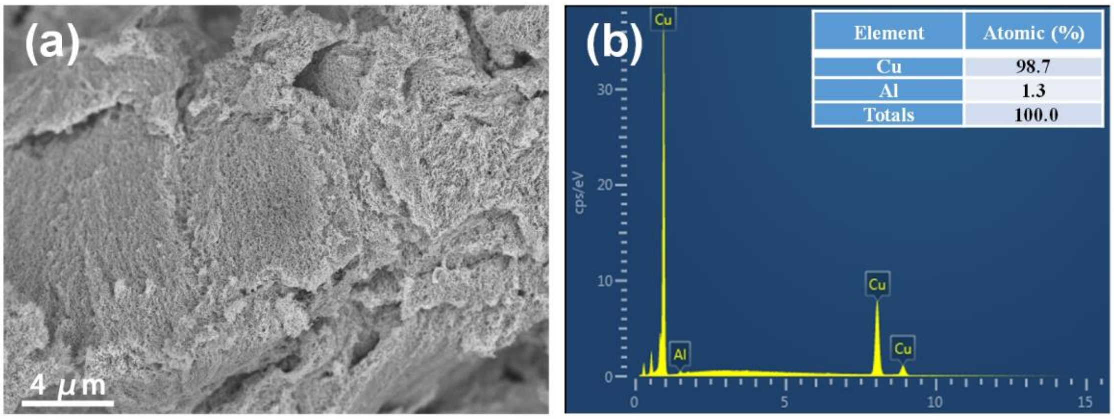

Fig. S3 (**a**) SEM image and (**b**) a typical EDX spectrum of the NP-Cu film

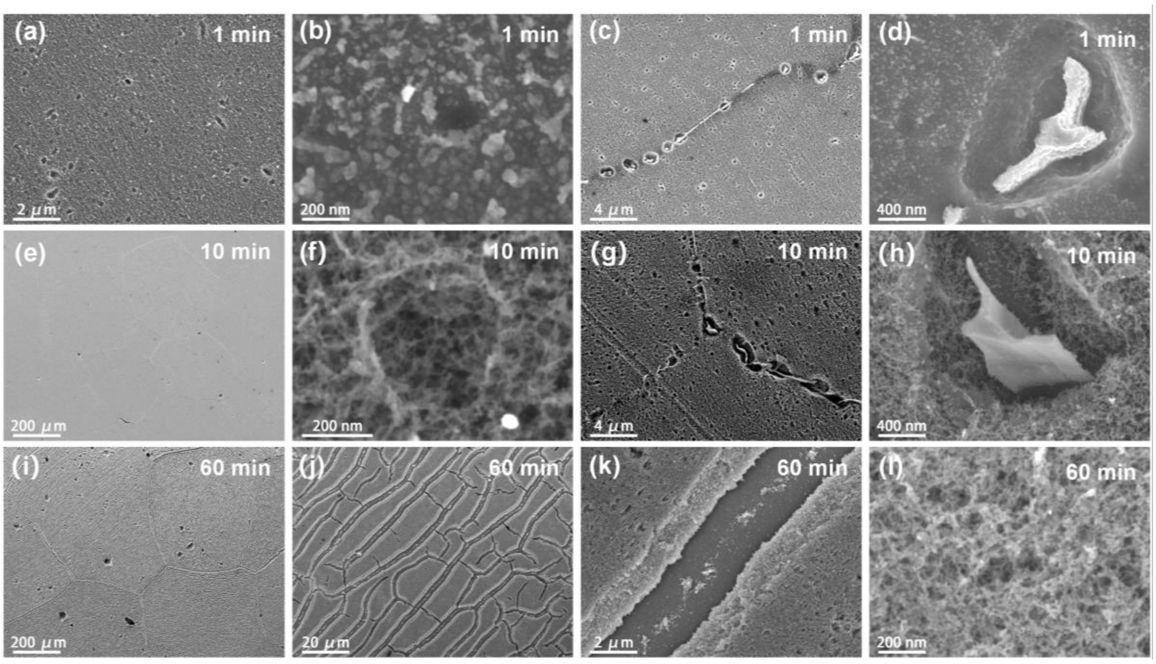

Fig. S4 Plan-view SEM images of the surface of the $Al_{98}Cu_2$ precursor dealloyed for different durations in the 0.5 M NaOH solution

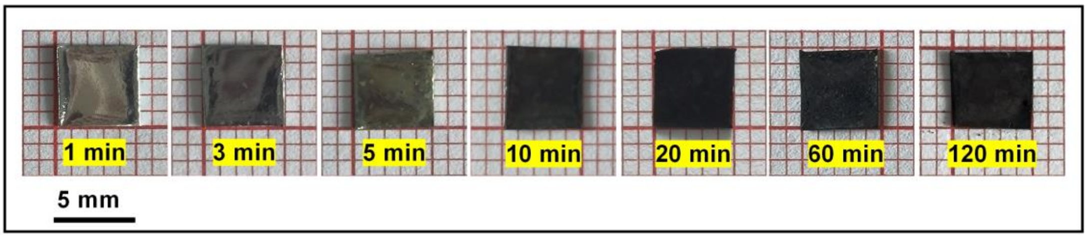

Fig. S5 Macrophotographs of the dealloyed $Al_{98}Cu_2$ samples at different dealloying times

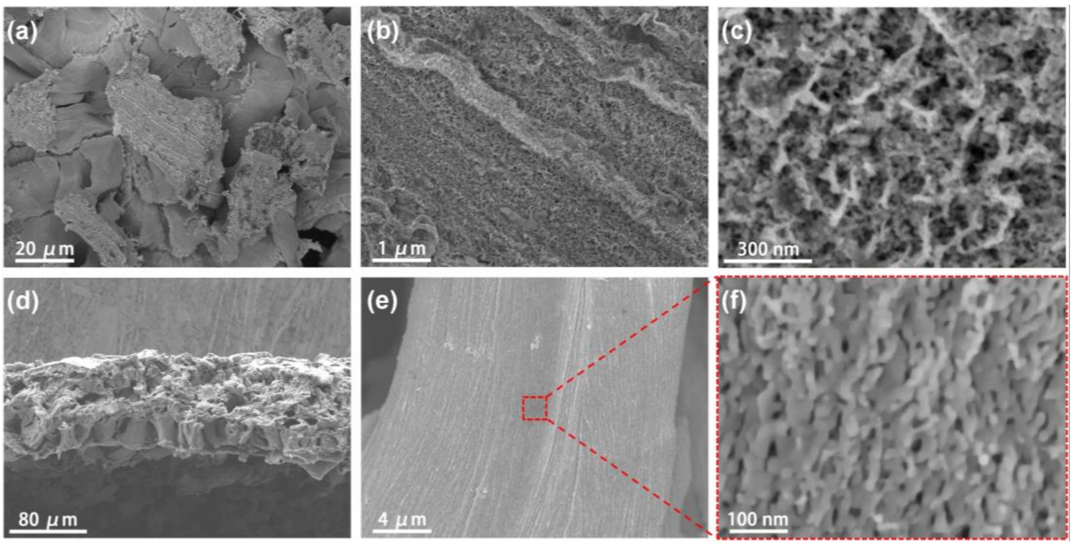

Fig. S6 (**a-c**) Plan-view and (**d-f**) cross-sectional SEM images of the NP-Cu film

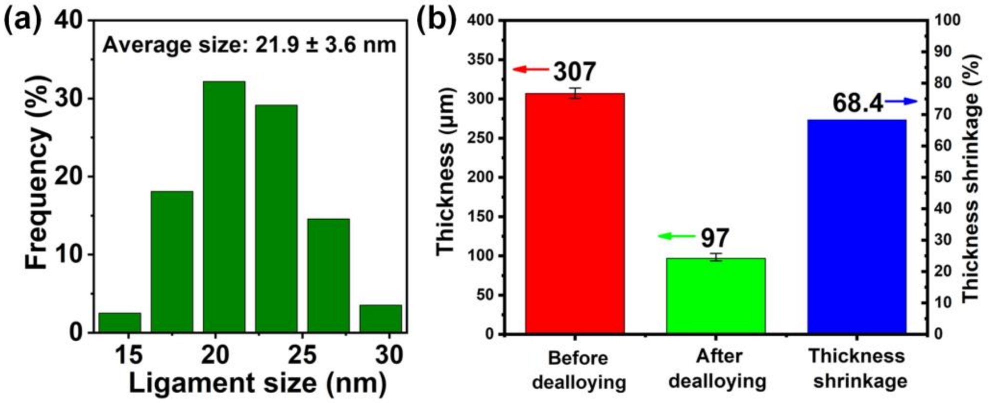

Fig. S7 (**a**) Ligament size distribution based upon the SEM images of the NP-Cu film. (**b**) Thickness change and thickness shrinkage of the NP-Cu film before and after dealloying

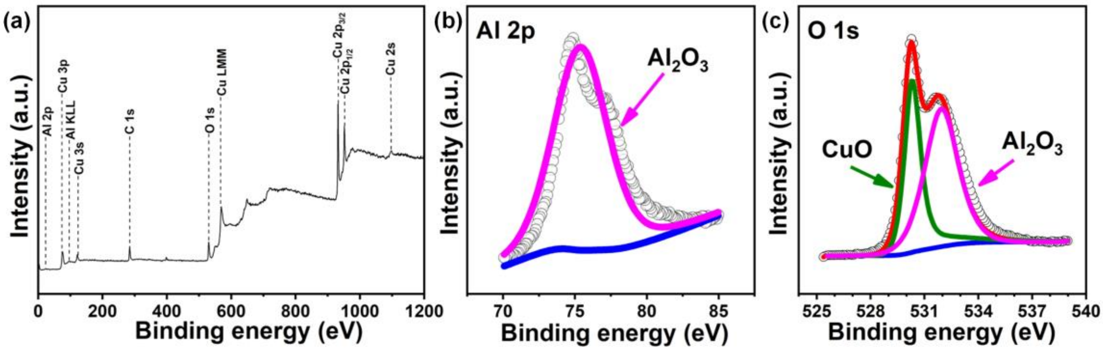

Fig. S8 (**a**) XPS survey spectrum of the NP-Cu film. (**b, c**) XPS spectra of (**b**) Al 2p and (**c**) O 1s for the NP-Cu film

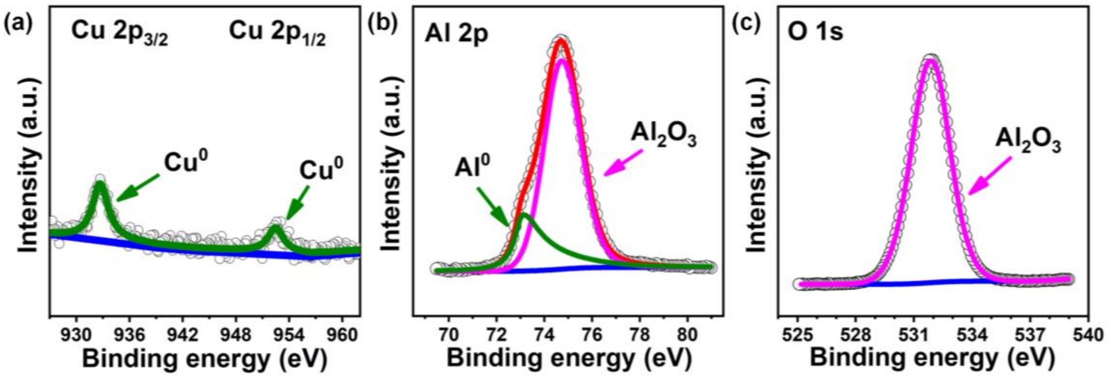

Fig. S9 (**a-c**) XPS spectra of (**a**) Cu 2p, (b) Al 2p and (**c**) O 1s for the $Al_{98}Cu_2$ precursor

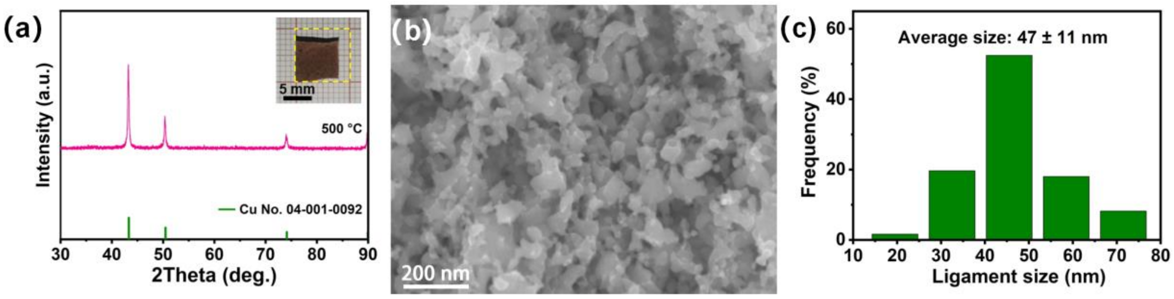

Fig. S10 (**a**) XRD pattern (Inset: macrophotograph, the yellow dashed line indicates the size before annealing), (**b**) SEM image and (**c**) ligament size distribution of the NP-Cu-500 film

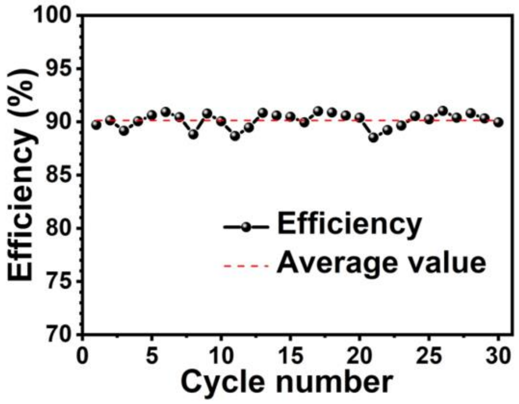

Fig. S11 Cycling test of the NP-Cu-500 film under 1 sun illumination

As can be seen from Fig. S11, the evaporation efficiency of the NP-Cu-500 film also fluctuates little in 30 cycles under 1 sun illumination. On the whole, the range of evaporation efficiency of the NP-Cu-500 film is always lower than that of the NP-Cu film (Fig. 5h). And the average evaporation efficiency of the NP-Cu-500 film in 30 cycles is 90.1%, smaller than that of the NP-Cu film (92.4%). The result shows that the NP-Cu-500 film has good stability and durability.

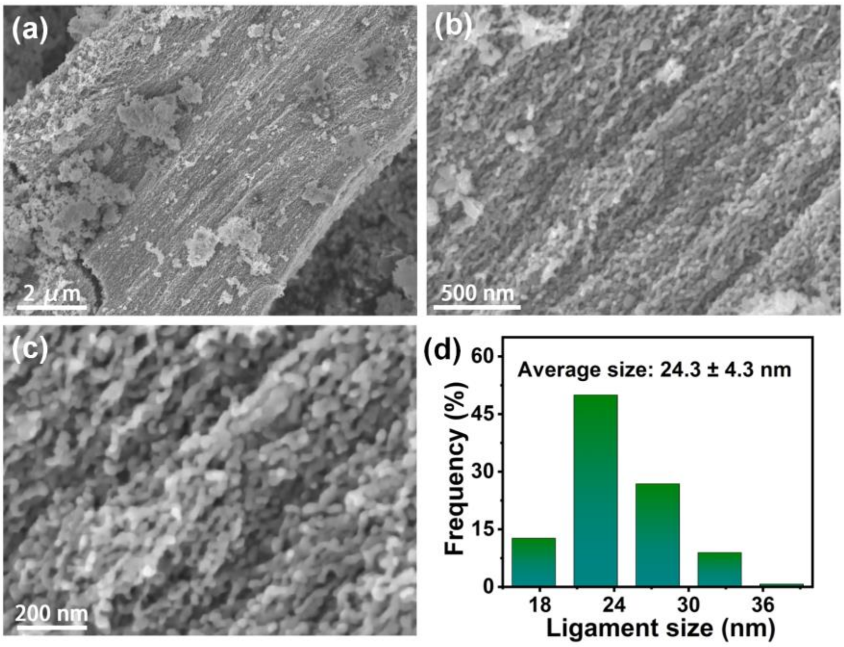

Fig. S12 (**a-c**) SEM images and (**d**) ligament size distribution of the NP-Cu film under 1 sun illumination after 30 cycles

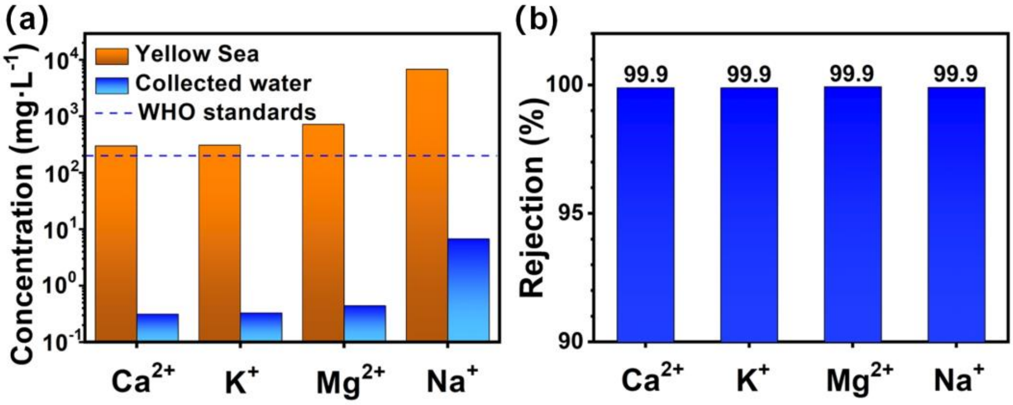

Fig. S13 (**a**) Concentrations of four metal ions in Yellow Sea and the collected clean water after desalination by the NP-Cu-500 film. (**b**) Corresponding ion rejection of real seawater sample after desalination

As can be seen from Fig. S13a, the ion concentration of Ca^{2+}, K^{+}, Mg^{2+} and Na^{+} in the seawater (Yellow Sea) decreases from 298.7, 307, 716, 6765 mg L^{-1} to 0.3, 0.3, 0.4, 6.7 mg L^{-1} respectively, which is greatly reduced after desalination. The collected water also meets the drinking water standards of the World Health Organization (WHO). And the NP-Cu-500 film also exhibits high ion rejections (Fig. S13b). These results indicate the NP-Cu-500 film has good seawater desalination ability.

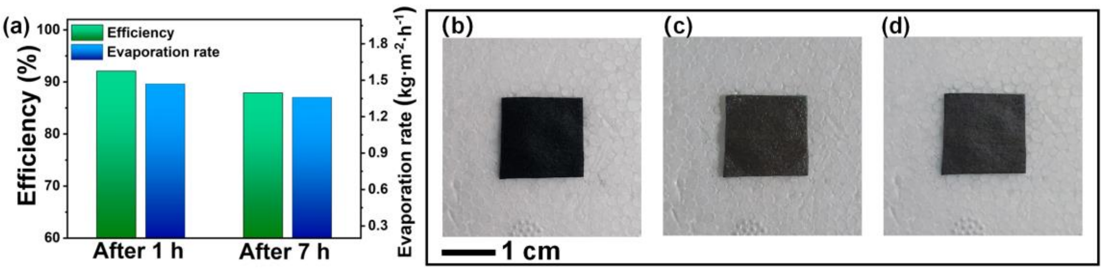

Fig. S14 (**a**) Evaporation efficiency and evaporation rate of the NP-Ag film after desalination for 1 and 7 h under 1 sun. (**b-d**) Photographs of the evaporation surface (**b**) before irradiation, (**c**) after 7 h irradiation under 1 sun and (**d**) after turning off light for 1 h

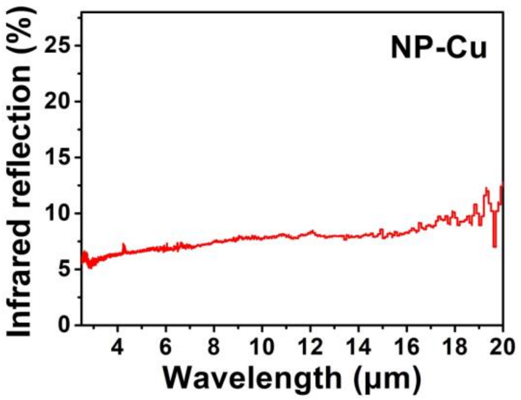

Fig. S15 Infrared reflection spectra of the NP-Cu film. After calculation, the emissivity of the NP-Cu is around 0.91

Table S1 Comparison of evaporation efficiency and cost of the NP-Cu film in this work and other typical noble metal-based photothermal materials. Recent metal prices come from the website www.kitco.com.

Materials	Metal cost ($/g)	Evaporation efficiency (%)	Refs.
NP-Au film	59.01	94.5 (under 1 sun)	[S1]
Au nanoparticles coated biomass foam	59.01	84 (under 1 sun)	[S2]
Au nanoparticles/ PBONF film	59.01	83 (under 1 sun)	[S3]
Au nanoparticles film	59.01	77.8 (under 4.5 sun)	[S4]
Au nanorod on carbon nanotube film	59.01	94 (under 5 sun)	[S5]
Au nanoparticles deposited sponge	59.01	90 (under 10 sun)	[S6]
Ag/PPy deposited substrates	0.69	92.6 (under 1 sun)	[S7]

NP-Ag film	0.69	92.6 (under 1 sun)	[S8]
Ag/polypyrrole hydrogels	0.69	88.7 (under 1 sun)	[S9]
Ag nanowires/rGO hydrogel	0.69	91 (under 1 sun)	[S10]
Pd coated wood	45.78	85 (under 10 sun)	[S11]
NP-Cu film	0.0087	92.9 (under 1 sun)	This work

The cost of Cu is inherently low, and its high porosity (94.8%) further reduces the cost. Moreover, from the above comparison, the evaporation efficiency of the NP-Cu film with wick structure in this work is comparable to or even better than that of other noble metal-based SSG systems. In the case of similar evaporation efficiency, the NP-Cu film has a great cost advantage.

S2 Calculation of Porosity

The porosity of the NP-Cu film was calculated by the following equation,

$$\varphi = \frac{V - V_{solid}}{V} \tag{S1}$$

where φ is the porosity, V_{solid} is the volume of the solid phase in the NP-Cu film, V is the total volume of the NP-Cu film. The volume of the solid phase can be calculated by measuring the mass of the NP-Cu film combined with the metal density of pure Cu (8.96 g cm^{-3}). Mass divided by density gives the volume of the solid phase. Taking one of the test data as an example, the mass of the NP-Cu film was 0.0073 g, thus the volume of the solid phase in the NP-Cu film was determined to be 8.15×10^{-4} cm^3. And the total volume of the NP-Cu film was 1.54×10^{-2} cm^3. Thus, the porosity of the NP-Cu film was determined to be 94.7%. The average of multiple sets of test results is 94.8%.

S3 Calculation of Heat Losses

In this work, the heat losses mainly include three ways, namely conduction heat loss, radiation heat loss and convection heat loss. The corresponding calculation process under one sun irradiation (1 kW m^{-2}) is as follows.

(1) Conduction heat loss

The heat conduction loss can be calculated by Fourier's law,

$$\eta_{cond} = \frac{kA_{section}\frac{\Delta T}{\Delta L}}{IA_{NPC}} \tag{S2}$$

where k is the thermal conductivity of the cotton pillar (0.04 W m^1 K^{-1}), $A_{section}$ is the sectional area of the cotton pillar (3.8×10^{-5} m^2), $\frac{\Delta T}{\Delta L}$ is the gradient of temperature

between the surface of the NP-Cu film and the bulk water, which was calculated to be around 329 K m^{-1}. I is 1 kW m^{-2} and A_{NPC} is the surface area of the NP-Cu film (4 cm^2). Thus, η_{cond} is 0.13%.

(2) Radiation heat loss

The radiation heat loss can be calculated by Stefan-Boltzmann law,

$$\eta_{rad} = \frac{\varepsilon \sigma A_{rad}(T_1^4 - T_0^4)}{I A_{NPC}} \tag{S3}$$

where ε is the emissive rate of the NP-Cu film (0.91, Fig. S15), σ is the Stefan-Boltzmann constant (5.67×10^{-8} W m^{-2} K^{-4}), A_{rad} is the radiative area (4 cm^2), T_1 is the average temperature of the evaporation surface in the stable state (37 °C), T_0 is the temperature of ambient environment near the NP-Cu film (28 °C). Thus, η_{rad} is 5.30%.

(3) Convection heat loss

The convection heat loss can be calculated by Newton's law of cooling,

$$\eta_{conv} = \frac{h A_{conv}(T_1 - T_0)}{I A_{NPC}} \tag{S4}$$

where A_{conv} is the convection area (4 cm^2), h is the convection heat transfer coefficient, which is assumed to be 5 W m^{-2} k^{-1} [S12]. Thus, the η_{conv} is 4.50%.

Based on the above results, the total heat loss of the NP-Cu film is around 9.93%.

Supplementary References

[S1] Y. Zhang, Y. Wang, B. Yu, K. Yin, Z. Zhang. Adv. Mater. 34(21), 2200108 (2022). https://doi.org/10.1002/adma.202200108

[S2] W. Fang, H. Chen, X. He, W. Li, W. Zhang, et al. J. Porous Mater. 28(6), 1655-1666 (2021). https://doi.org/10.1007/s10934-021-01112-1

[S3] M. Chen, Y. Wu, W. Song, Y. Mo, X. Lin, et al. Nanoscale 10(13), 6186-6193 (2018). https://doi.org/10.1039/C8NR01017J

[S4] Y. Liu, S. Yu, R. Feng, A. Bernard, Y. Liu, et al. Adv. Mater. 27(17), 2768-2774 (2015). https://doi.org/10.1002/adma.201500135

[S5] Y. Yang, X. Yang, L. Fu, M. Zou, A. Cao, et al. ACS Energy Letters 3(5), 1165-1171 (2018). https://doi.org/10.1021/acsenergylett.8b00433

[S6] Y. Liu, Z. Liu, Q. Huang, X. Liang, X. Zhou, et al. J. Mater. Chem. A 7(6), 2581-2588 (2019). https://doi.org/10.1039/C8TA10227A

[S7] Y. Xu, J. Ma, Y. Han, H. Xu, Y. Wang, et al. Chem. Eng. J. 384, 123379 (2020). https://doi.org/10.1016/j.cej.2019.123379

[S8] B. Yu, Y. Wang, Y. Zhang, Z. Zhang. Nano Res., (2022). https://doi.org/10.1007/s12274-022-5068-x

[S9] C. Xiao, W. Liang, Q.-M. Hasi, L. Chen, J. He, et al. Mater. Today Energy 16, 100417 (2020). https://doi.org/10.1016/j.mtener.2020.100417

[S10] C. Liu, C. Cai, F. Ma, X. Zhao, H. Ahmad. J. Colloid Interface Sci. 560, 103-110 (2020). https://doi.org/10.1016/j.jcis.2019.10.055

[S11] M. Zhu, Y. Li, F. Chen, X. Zhu, J. Dai, et al. Adv. Energy Mater. 8(4), 1701028 (2018). https://doi.org/10.1002/aenm.201701028

[S12] L. Momenzadeh, B. Moghtaderi, O. Buzzi, X. Liu, S. W. Sloan, et al. Comput. Mater. Sci. 141, 170-179 (2018). https://doi.org/10.1016/j.commatsci.2017.09.033
